# Three-Dimensional Electro-Fenton System with CuFe_2_O_4_-Loaded Granular Activated Carbon as the Catalytic Particle Electrode for Removal of Bisphenol A

**DOI:** 10.3390/nano16120722

**Published:** 2026-06-11

**Authors:** Sheng Tao, Zhang Luo, Defeng Kong, Yifan Chai, Shenglong Kuai, Huilai Liu, Cheng Yin, Xing Chen

**Affiliations:** 1 School of Resources and Environmental Engineering, Hefei University of Technology, Hefei 230009, China; 19856125698@163.com (S.T.); cyf_010906@163.com (Y.C.); 2 Key Lab of Aerospace Structural Parts Forming Technology and Equipment of Anhui Province, Institute of Industry and Equipment Technology, Hefei University of Technology, Hefei 230009, China; 3 CCCC Yangtze River Construction Development Group Co., Ltd., Chongqing 400700, China; 602025250036@smail.nju.edu.cn; 4 Anhui Haoyue Ecological Technology Co., Ltd., Hefei 230071, China; kdf_001.student@sina.com; 5 School of Resource and Environmental Engineering, Anhui Water Conservancy Technical College, Hefei 231603, China; ksl@ahsdxy.edu.cn (S.K.); liuhuilai_1996@163.com (H.L.)

**Keywords:** three-dimensional electro-Fenton, activated carbon particle electrode, bimetallic synergistic effect, Bisphenol A, wastewater treatment

## Abstract

Bisphenol A (BPA) is a typical endocrine disruptor that poses a significant threat to ecosystems. Therefore, it is crucial to develop an efficient and environmentally friendly degradation technology. In this study, a novel bimetallic oxide-loaded GAC (Granulated Activated Carbon) particle electrode (CuFe_2_O_4_@GAC) was designed and applied to a three-dimensional electro-Fenton (3D-EF) system for efficient removal of BPA. The bimetallic synergistic effect of Cu and Fe was used to promote the Fenton reaction and enhance the efficiency of hydroxyl radical ·OH generation. The results show that under conditions of 20 g/L CuFe_2_O_4_@GAC, pH = 3, 10 mA/cm^2^, and an electrode spacing of 2.0 cm, a BPA removal rate of over 93% (20 mg/L) was achieved within 45 min. The prepared CuFe_2_O_4_@GAC exhibits good stability, maintaining an 86.2% BPA degradation rate over five cycle experiments. The catalytic mechanism and degradation pathways were further analyzed through characterization methods such as radical quenching experiments, XPS analysis, EPR, and LC-MS detection. Radical quenching experiments confirmed that ·OH radicals play a significant role in the decomposition of BPA. Based on the identification of intermediates, a possible decomposition pathway for BPA was proposed. Toxicity analysis indicated that the toxicity of most intermediates was significantly lower than that of BPA. This work provides an efficient and energy-saving strategy for BPA removal.

## 1. Introduction

Bisphenol A (BPA) is an organic compound widely used in industrial production, belonging to phenolic substances. Its core characteristic is its good stability and plasticity, so it is widely used to make plastics, resins, and other materials that are closely related to daily necessities, but it also attracts widespread attention because of its potential health risks [1]. However, the traditional water treatment technology cannot meet the needs of BPA wastewater treatment with different concentrations and different water qualities, so it is very important to develop efficient and cost-saving advanced treatment technology to treat BPA wastewater.

As a highly promising technological approach, electrochemical advanced oxidation processes (EAOPs) can effectively degrade and remove organic pollutants [2]. Their mechanism of action is based on two key steps: (1) the electro-adsorption of pollutants onto the surface of granular activated carbon (GAC), and (2) the in situ oxidative degradation of organic contaminants. This process is mainly mediated by hydroxyl radicals (·OH), whose standard redox potential (E∘(·OH/H_2_O) = 2.80 V vs. SHE) is second only to that of fluorine, endowing them with extremely strong non-selective oxidizing capacity. Electro-Fenton (EF) technology cleverly combines the merits of electrochemical processes and the conventional Fenton reaction. Unlike the exogenously added in conventional Fenton systems, EF technology realizes the in situ synthesis of hydrogen peroxide through two-electron oxygen reduction reactions occurring on the cathode surface. Notably, the in situ-generated H_2_O_2_ serves as a critical precursor for producing highly reactive hydroxyl radicals, and it also acts as a strong oxidant with exceptional oxidation capability, which can directly oxidize partial organic contaminants in aqueous solutions or synergistically enhance the degradation efficiency of target pollutants [3,4,5]. In the electro-Fenton (EF) system [6,7], the pathways for ·OH generation include the Fenton reaction, where H_2_O_2_ is catalyzed by Fe^2+^ to produce ·OH; anodic oxidation, where water molecules are discharged on the electrode surface to directly generate M(·OH) when using inert anodes with high oxygen evolution overpotential (e.g., boron-doped diamond, BDD) [8,9,10]; cathodic reduction, where O_2_ undergoes two-electron reduction to continuously generate H_2_O_2_ in situ; and catalyst regeneration, where Fe^3+^ is reduced to Fe^2+^ at the cathode to maintain the catalytic cycle [5].

Three-dimensional electrocatalytic technology is a heterogeneous catalytic oxidation system based on the traditional two-dimensional flat electrode system, which is constructed by introducing particle electrodes [11,12,13]. Combining this technology with the EF reaction, a three-dimensional EF system can be constructed. With the advantages of efficient mass transfer and active sites of three-dimensional electrocatalysis, the generation efficiency of hydroxyl radicals in EF reaction is strengthened, and the pain points of poor mass transfer and insufficient active sites in traditional two-dimensional electro-Fenton are solved. Therefore, the development of particle electrodes with high catalytic activity is the key to achieving efficient degradation in the process. Yang et al. adopted Fe-modified sludge biochar (Fe@SBC) as the particle electrode, and the Fe@SBC prepared at 800 °C exhibited excellent capacities for H_2_O_2_ electrogeneration and activation [14]. Various 3D particle electrode and electro-Fenton systems have been widely developed for organic wastewater treatment, but most still suffer from inherent limitations, including a narrow acidic pH working window, slow redox kinetics of single metal sites, low TOC mineralization capacity, and high energy consumption (Table 1). In addition, many carbon-based electrodes face the dilemma between electrical conductivity and structural defects, and most reported materials exhibit obvious activity decay and metal leaching during cycles [15,16,17,18].

Due to its high specific surface area, developed pore structure, and good electrical conductivity, activated carbon (AC) is commonly utilized as a particle electrode in three-dimensional electrode systems. The Fe element is the core catalytic component of the Fenton reaction cycle, which can efficiently catalyze the decomposition of hydrogen peroxide to generate hydroxyl radicals. As a synergistic catalyst, the Cu element can not only accelerate the reduction of Fe^3+^ to Fe^2+^, but also improve the efficiency of the catalytic cycle. In some studies, it has been proven that loading metal elements on the surface of particle electrodes can significantly improve the catalytic activity [19,20,21]. Comparatively speaking, the GAC particle electrode of bimetallic oxide combines the advantages of the synergistic effect of a porous GAC conductive skeleton and a bimetallic spinel. Spinel structure has the inherent advantages of the redox cycle of bimetal, with a stable crystal structure and highly dispersed metal sites.

Therefore, in this study, a carbon-based bimetallic oxide particle electrode (CuFe_2_O_4_@GAC) was simply synthesized by the impregnation hydrothermal method, and a cuboid electrolytic cell was designed. Subsequently, the simulated Bisphenol A wastewater solution was degraded by a three-dimensional electro-Fenton oxidation technology consisting of CuFe_2_O_4_@GAC particle electrode material and an electrolytic cell.

## 2. Materials and Methods

### 2.1. Chemicals

Graphite flake was purchased from China Dongguan Tianwang Graphite Products Co., Ltd., and columnar activated carbon (AC) was purchased from Quanzhou Yuansen Environmental Protection Technology Co., Ltd. in China. The AC parameters are as follows: iodine value is about 800 mg/g, 4.0 mm.

Copper nitrate trihydrate (Cu(NO_3_)_2_·3H_2_O), iron nitrate nonahydrate (Fe(NO_3_)_3_·9H_2_O), sodium hydroxide (NaOH), sodium sulfate (Na_2_SO_4_), concentrated nitric acid (HNO_3_), concentrated sulfuric acid (H_2_SO_4_), acetonitrile, anhydrous ethanol, anhydrous methanol, tert-butyl alcohol (TBA), and p-benzoquinone. All chemical reagents were utilized directly without additional purification, and all aqueous solutions were prepared using deionized water.

### 2.2. Preparation of Catalytic Particle Electrodes

The synthesis of CuFe_2_O_4_@GAC particle electrodes mainly includes the modification and loading of the activated carbon surface. First, 0.1 mol of copper nitrate trihydrate and 0.1 mol of ferric nitrate nonahydrate (Cu(NO_3_)_2_·3H_2_O and Fe(NO_3_)_3_·9H_2_O) were placed into a beaker with 15 mL of deionized water, and stirred for 15 min to mix them thoroughly [22,23]. Then, 8 g of granular activated carbon, which had been acid-treated and rinsed to neutrality, was added to the beaker and ultrasonically dispersed for 15 min. Subsequently, 0.2 mol of NaOH was slowly added to 60 mL of deionized water under stirring until fully dissolved. This NaOH solution was then slowly poured into the beaker; after a large amount of dark flocculent precipitate was generated, the mixture was stirred uniformly and left to stand for 15 min. The resulting mixture was transferred into a 100 mL reaction kettle and subjected to hydrothermal treatment at 180 °C in an oven for 15 h [24,25,26]. After cooling, the prepared particle electrodes were taken out, washed with deionized water until the pH reached neutrality, and dried for subsequent use.

### 2.3. Characterization

The concentration of BPA was detected by liquid chromatography. The morphological features of the materials were observed and documented by means of a high-resolution field emission scanning electron microscope (FE-SEM, Regulus 8230, Hitachi, Tokyo, Japan). High-resolution transmission electron microscopy (HR-TEM) characterization was carried out using a Talos F200X G2 microscope (Thermo Fisher Scientific, Waltham, MA, USA), where imaging results were acquired at an accelerating voltage of 200 kV. X-ray photoelectron spectroscopy (XPS) data were gathered with an ESCALAB250Xi XPS spectrometer (Thermo Fisher Scientific, USA), while the XRD patterns of the materials were measured using a D/MAX2500VL/PC X-ray diffractometer (Rigaku, Tokyo, Japan). The hybridization state of the carbon materials was examined via a micro-Raman spectrometer. Based on the Brunauer–Emmett–Teller (BET) theory, the specific surface area of the samples was computed using an Autosorb-IQ3 instrument (Quantachrome Instruments, Boynton Beach, FL, USA). The concentration of leached metal ions in the solution was detected by inductively coupled plasma mass spectrometry (ICP-MS). Electron paramagnetic resonance (EPR) tests were performed on a JES-FA200 spectrometer (JEOL, Tokyo, Japan), and the TOC removal rate was quantified with a Multi N/C 3100 TOC analyzer (Analytik Jena, Jena, Germany). Additionally, the analysis of intermediate products generated during Bisphenol A (BPA) degradation was achieved using a liquid chromatography–quadrupole electrostatic field orbitrap mass spectrometer (LC-MS, Thermo Fisher Scientific, USA).

### 2.4. Experimental Methods

The heterogeneous electrocatalytic reaction of 3D-EF was carried out in a 150 mL electrolytic cell. The anode and cathode were composed of 1 mm thick graphite sheet, with an area of 10 cm^2^. An activated carbon particle electrode (adsorbing saturated BPA) was placed between the anode and cathode. The target pollutant was set as BPA, with a concentration of 20 mg/L, and the electrolyte employed was 0.1 mol/L sodium sulfate. The pH value of the reaction system was adjusted using 3 mM sulfuric acid and 1 mM sodium hydroxide, and the entire reaction process was conducted under the condition of continuous oxygen aeration (0.1 L/min). The key factors include the dosage of CuFe_2_O_4_@GAC particle electrode (10–30 g/L), current intensity (0.05–0.3 A), electrode spacing (20 mm, 25 mm, and 30 mm), initial pH value of electrolyte (3, 5, 7, and 9), and reaction time for removing Bisphenol A (0–45 min). In order to verify the practical application potential of the electrocatalytic process, synthetic wastewater was prepared from tap water and surface water to simulate the wastewater scene. Subsequently, the process was extended to the treatment of real wastewater samples to evaluate its degradation efficiency more comprehensively.

### 2.5. Electrochemical Tests

Electrochemical signals were detected in a 10 mL electrolytic cell adopting a conventional three-electrode configuration, which consisted of a platinum wire, a glassy carbon electrode (Drop-casting Method), and an Ag/AgCl electrode. After being ground into a uniform powder, the catalyst was dropped onto the glassy carbon electrode and dried for backup use. The acquired sensor data were further processed by a computer-controlled CHI760E potentiostat integrated with an electrochemical workstation.

## 3. Results and Discussion

### 3.1. Structural and Physical Characterization

**Figure 1:**
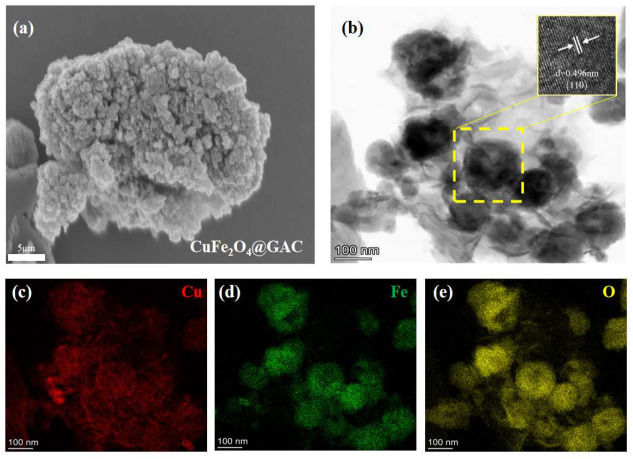
(**a**) SEM morphologies of CuFe_2_O_4_@GAC. (**b**) HRTEM image of CuFe_2_O_4_@GAC. (**c**–**e**) Elemental mapping images of the CuFe_2_O_4_@GAC.

As shown in (Figure S1), the surface of pure activated carbon (GAC) without metal oxide loading is smooth and flat, and there is no obvious granular protrusion or impurity attachment, which indicates that the original GAC has a clean surface base and provides a good bearing condition for subsequent metal oxide loading. High-resolution SEM clearly reveals the uniform dispersion of linear CuO, Fe_2_O_3_, and agglomerated CuFe_2_O_4_ on the activated carbon surface (Figure S1). The morphological distribution and particle size of CuFe_2_O_4_ nanoparticles were characterized by means of scanning transmission electron microscopy (TEM) coupled with energy-dispersive X-ray spectroscopy (Figure 1b). The results indicate that the CuFe_2_O_4_ nanoparticles are arranged on the GAC surface. The presence of Fe, Cu, and O was confirmed by elemental mapping data (Figure 1c–e).

**Figure 2:**
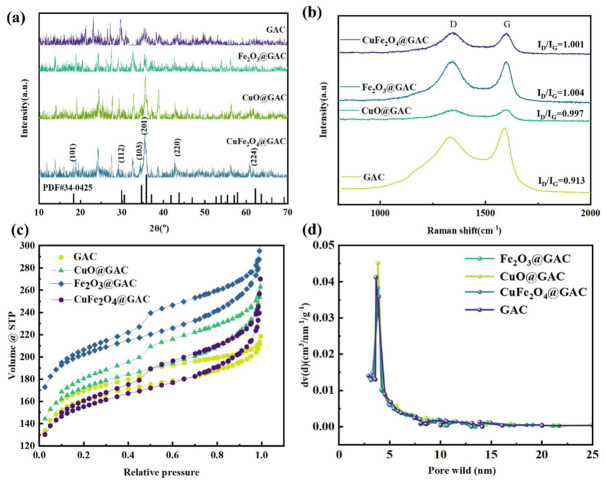
(**a**) XRD patterns, (**b**) Raman spectra, (**c**) N_2_ adsorption isotherms, and (**d**) pore size distribution of GAC, Fe_2_O_3_@GAC, CuO@GAC, and CuFe_2_O_4_@GAC catalysts.

X-ray diffraction (XRD) patterns are shown in Figure 2a. The diffraction peaks of CuFe_2_O_4_@GAC were consistent with those of pure CuFe_2_O_4_ (PDF#34-0425), indicating the successful synthesis of CuFe_2_O_4_. The samples have obvious diffraction peaks at 2θ = 18.51, 30.17, 35.64, 37.16, 43.09, 57.00, and 62.77, which correspond to (111), (220), (311), and (224) of CuFe_2_O_4_, respectively. In addition, there is no obvious impurity peak in the spectrum, and the diffraction peak is sharp and symmetrical, which shows that CuFe_2_O_4_ has high crystallinity and a complete lattice structure.

The Raman spectra of four materials are shown in Figure 2b. The characteristic peaks of the D band and G band appeared at 1350 cm^−1^ and 1591 cm^−1^, corresponding to disordered carbon and graphitized carbon, respectively. The intensity ratio of the D band to the G band (I_D_/I_G_) can be used to characterize the graphitization degree or disorder degree of carbon materials: a higher I_D_/I_G_ ratio indicates a higher disorder degree and more defects, which is beneficial to enhancing the catalytic activity of the materials [27]. The I_D_/I_G_ ratios of CuO@GAC, Fe_2_O_3_@GAC, and CuFe_2_O_4_@GAC were calculated to be 0.997, 1.004, and 1.001, respectively. Compared with pristine GAC, the three synthesized composites exhibited increased I_D_/I_G_ ratios, implying a higher disorder degree and more defects. Raman analysis indicates the material retains a favorable graphitized carbon framework and contains abundant structural defects, both jointly supplying sufficient active sites for catalytic reactions. Although the ratio of Fe_2_O_3_@GAC is the highest, indicating that more structural defects can be used as anchoring sites, the catalytic activity is mainly determined by the intrinsic properties of the supported metal oxides. In contrast, the Cu-Fe oxide loading shows a more uniform and well-dispersed structure, which provides a larger electrochemically active surface area and more accessible bimetallic active sites. Despite its low carbon defect density, it still has excellent catalytic performance.

The test results of N_2_ adsorption–desorption isotherms (Figure 2c) systematically revealed the regulatory effect of different modification treatments on the pore structure of carbon materials. The specific surface area of CuFe_2_O_4_@GAC shows a slight increase compared with that of raw, unmodified granular activated carbon. The specific surface area rises from 31.946 m^2^/g of pristine GAC to 39.995 m^2^/g after metal oxide loading, presenting a moderate growth in pore structure. The high specific surface area of CuFe_2_O_4_@GAC indicates that the metal oxides are uniformly dispersed and provide a large number of new surfaces, but the pore volume is low, indicating that these metal oxides are deposited inside the pores and fill part of the pore space. In contrast, the pore volumes of CuO@GAC and Fe_2_O_3_@GAC are higher, indicating that the single metal oxides may be mainly loaded on the outer surface, or the formed particles are larger (linear) and do not enter the pores (Table S2). This phenomenon indicates that the loading of metal oxide nanoparticles on the GAC surface did not damage its main pore structure; instead, it optimized the fine structure of the pore channels through moderate filling and surface modification of nanoparticles in partial macroporous or mesoporous channels, thus achieving the improvement of specific surface area and precise regulation of pore size (Figure 2d). Further analysis of the pore size distribution curves showed that the activated carbon loaded with metal oxides contained a large number of mesopores. The mesopore size (3.8 nm) is much larger than the BPA molecular size (1.0 nm), and the pollutants can freely diffuse into the pores. The mesopores provide mass transfer channels for H_2_O_2_ and ·OH to reach the active sites efficiently. The mesoporous structure can achieve the synergy of adsorption enrichment and in situ catalytic degradation, avoid the pore channel blocking problem that is prone to occur in micropores, and thereby accelerate the catalytic reaction rate. In addition, the mesoporous structure can enhance the adsorption effect on organic pollutant BPA, enrich the pollutant near the active sites, and then improve the efficiency of electro-Fenton oxidative degradation.

**Figure 3:**
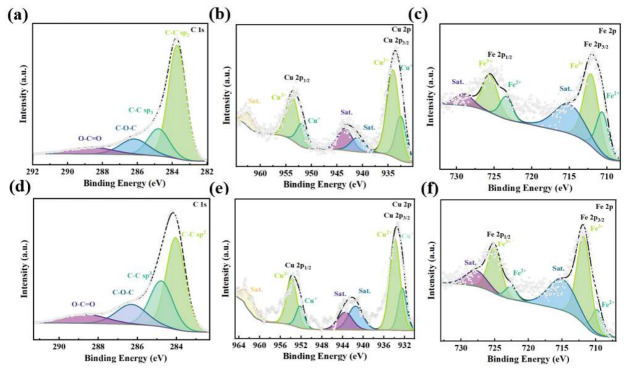
XPS spectra of (**a**,**d**) C 1s, (**b**,**e**) Cu2p, and (**c**,**f**) Fe 2p of the fresh and used CuFe_2_O_4_@GAC.

XPS full-spectrum scanning and quantitative analysis show that CuFe_2_O_4_@GAC mainly contains four characteristic elements (Figure S4c,d), C, O, Fe and Cu, and there is no significant difference in the total amount of elements: the atomic proportion of element C is stable at 72.8–73.0%, and the main peak of C 1s is always at 284.78–284.80 eV (Figure 3a), corresponding to the C-C/C-H bond of GAC matrix. The atomic ratio of Fe (0.92–0.93%) to Cu (0.70–0.71%) is always close to 2:1, and the atomic percentage change is less than 0.1%, and it is proven that CuFe_2_O_4_ nanoparticles are stably bound to GAC. In the Fe 2p spectrum, the main peak of Fe 2p before the reaction is located at 711.5 eV, and the ratio of Fe^2+^/Fe^3+^ is 0.58 (Figure 3c). After the reaction, the peak-to-peak high binding energy of Fe 2p shifted to 712.2 eV, and the ratio of Fe^2+^/Fe^3+^ is 0.12. This is because Fe^2+^ referentially reacted with H_2_O_2_ to generate Fe^3+^, so the final proportion of Fe^2+^ was lower than the initial value. In the spectrum of Cu 2p, before the reaction, the Cu 2p main peak is stable at 934.6 eV, and the ratio of Cu^+^/Cu^2+^ is 0.21 (Figure 3b). After the reaction, the ratio of Cu^+^/Cu^2+^ is 0.18. The proportion of Cu^+^ is relatively decreased, because Cu^+^ can also be used as the active site of the Fenton reaction and participate in the formation of ·OH. In the O 1s spectrum, the ratio of the peak area of hydroxyl oxygen (O-H, 531.5 eV) before the reaction was 38.86%, and it rose to 44.68% after the reaction. In the O1s spectrum before the reaction, the Ov peak intensity is significant, indicating that the surface of CuFe_2_O_4_@GAC is rich in hydroxyl oxygen and oxygen vacancies. These species are the key active sites for H_2_O_2_ activation in the electro-Fenton system. The high Ov content provides a structural basis for the efficient generation of ·OH (Figure S4a,b). After the reaction, the relative intensity of the Ov peak decreased, and the relative intensity of the O_L_ peak increased. During the reaction, the surface hydroxyl group (M-OH) was consumed and partially converted into lattice oxygen (M-O-M). This verifies that hydroxyl oxygen is directly involved in the ·OH generation reaction. Consistent with the previous characterization results, the existence of CuFe_2_O_4_ oxide was confirmed.

### 3.2. BPA Removal Efficiencies in the 3D-EF

**Figure 4:**
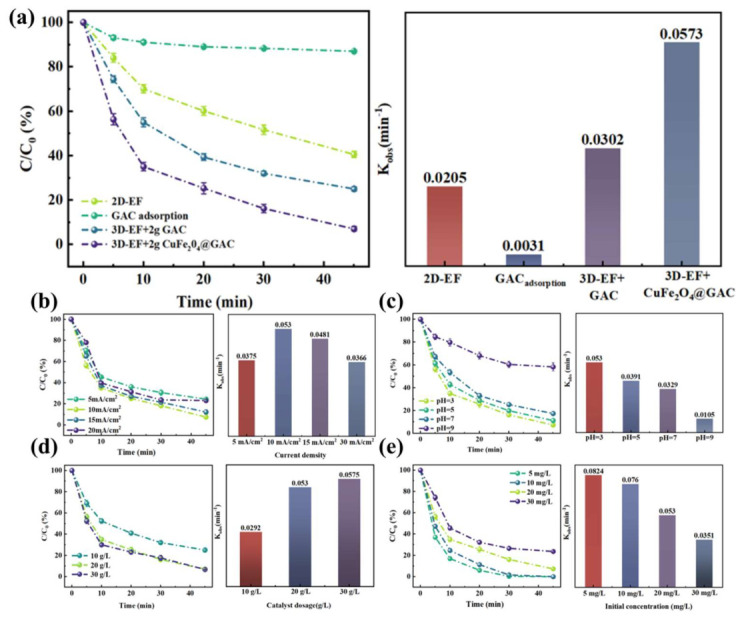
(**a**) Degradation of BPA in different systems. (**b**–**e**) Effects of current densities, different pH, particle electrode dosage, and different BPA initial concentrations on the degradation of BPA in the 3D-EF system. Unless otherwise specified, the reaction conditions are as follows: initial concentration of BPA = 20 mg/L; dosage of particle electrode = 20 g/L; current density = 10 mA/cm^2^; and initial pH = 3.

The primary objective of this research is to explore the degradation efficiency of the synthesized catalyst towards organic pollutants in the target system. To explore the enhancement impact of metal oxide loading modification on the performance of particle electrodes and the optimal parameters for BPA degradation in the EF system, unloaded granular activated carbon (GAC) and three kinds of metal oxide-loaded particle electrodes were chosen for experimental studies. Preliminary experiments examined the performance of different catalysts under fixed conditions (particle electrode dosage: 20 g/L, pH = 3, BPA concentration: 20 mg/L). The findings indicated that the degradation efficiency achieved by the three-dimensional (3D) electrode configuration was substantially higher, approximately twice that of the conventional two-dimensional (2D) electrode setup (Figure 4b).

In addition, this study systematically investigated the effects of several experimental operating parameters on the degradation process, including pH, particle electrode dosage, current density, and initial BPA concentration. Under a unified reaction system, comparative results showed that the BPA degradation efficiency without GAC addition was only 59.5%, and that with unloaded GAC addition reached 75%. Among the tested catalysts, CuFe_2_O_4_@GAC exhibited the optimal catalytic activity due to its unique spinel structure and synergistic effect with GAC, achieving a degradation rate of 93%. As a core regulatory factor, the solution pH in the range of 3.0–9.0 showed that the degradation rate peaked at 93% at pH = 3.0 (Figure 4c); the acidic environment at this pH favored the Fe^2+^/Fe^3+^ cycle and ·OH generation. In contrast, when the pH increased to 9.0, the degradation rate dropped to 41.7%, mainly because Fe^3+^ tends to form hydroxide precipitates under alkaline conditions, ·OH undergoes self-quenching, and the reaction activity of BPA molecules decreases. The degradation effect remained stable when the initial BPA concentration was in the range of 5.0–10.0 mg/L (Figure 4d); beyond this threshold, the degradation rate decreased significantly, which was attributed to the competition between a large number of intermediate products and BPA for ·OH, occupation of active sites, and excessive consumption of free radicals at high concentrations [3,28,29]. The degradation rate remained stable at 93% within a current density range of 10–15 mA/cm^2^ (Figure 4e). This stability can be attributed to the excessive current density causing water heating, which triggers the non-productive decomposition of H_2_O_2_ into H_2_O instead of catalyzing the formation of ·OH. Conversely, increasing the particle electrode dosage from 1.0 to 3.0 g (Figure 4d) resulted in the continuous enhancement of both degradation efficiency and kinetic rate, owing to the increased number of active sites and improved ·OH generation efficiency. However, the effect showed no significant change when the dosage exceeded 2.0 g, and excessive addition would lead to an increase in hydroxide precipitates due to ion leaching, resulting in a decrease in the kinetic rate.

In summary, metal oxide loading modification can improve the catalytic performance of activated carbon particle electrodes, and CuFe_2_O_4_@GAC is the preferred catalyst for BPA degradation in the 3D-EF system. The optimal reaction conditions are pH = 3.0, an initial BPA concentration of 20 mg/L, and a particle electrode dosage of 20 g/L. This study provides an important experimental basis and offers practical technical support for the efficient treatment of wastewater containing BPA.

### 3.3. Degradation Mechanism

**Figure 5:**
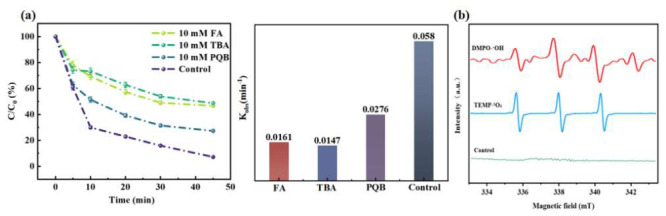
(**a**) Radical scavenging experiment of 3DEF-CuFe_2_O_4_@GAC. (**b**) DMPO-·OH and TEMP-^1^O_2_ in 3DEF system.

To elucidate the free radical degradation mechanism in the 3D-EF system, tert-butanol (TBA), p-benzoquinone (PQB), and furfuryl alcohol (FFA) were adopted as selective radical scavengers with a uniform concentration of 10 mM (Figure 5a). Each scavenger possesses specific selectivity toward different reactive oxygen species. TBA selectively captures ·OH, FFA quenches ^1^O_2_, and PQB mainly eliminates ·O_2_^−^.


(1) $$Contribution_{ROS}=(k_{blank}-k_{scavenger})/k_{blank}\times 100\%$$

Without scavengers, the BPA degradation efficiency reached 93%. Upon addition of 10 mM TBA and 10 mM FFA, the degradation efficiency decreased to 50.3% and 52.3%, respectively. In comparison, the degradation efficiency only dropped to 72.6% when 10 mM PQB was introduced. By calculating the reaction kinetic constants of various ROS quenching experiments, the contribution of ROS in the reaction system was qualitatively analyzed according to the free radical contribution formula. There is a mutual transformation between the active species: ·O_2_^−^ will undergo a disproportionation reaction to generate ^1^O_2_, and the electrons obtained by ^1^O_2_ will also be reduced to ·O_2_^−^. Secondly, the reaction of ·O_2_^−^ with H_2_O_2_ also produces ·OH. Therefore, ·OH and ^1^O_2_ were the main active species, followed by ·O_2_^−^ (Figure 5b).

Combined with EPR results (Figure 5b), the typical 1:2:2:1 quadruple signal of the DMPO-·OH adduct and 1:1:1 triple signal of the TEMP-^1^O_2_ adduct were clearly detected [30,31,32]. This further confirms that ·OH dominates the degradation process, followed by ^1^O_2_, and ·O_2_^−^ plays an auxiliary role in this reaction system.

Based on the experimental results, a proposed schematic of the system’s potential electrocatalytic degradation mechanism is illustrated in Figure S7. The CuFe_2_O_4_@GAC composite exhibits low electron transfer resistance and a high electron transfer rate, which collectively facilitate charge transfer and favor redox reactions. During the initial reaction stage, oxygen molecules adsorbed from the solution diffuse to the active sites on the electrode surface, where they are reduced in situ to hydrogen peroxide (H_2_O_2_) via a two-electron pathway.

The electron-rich low-valent Fe^2+^ in granular activated carbon (GAC) serves as the main active site for activating H_2_O_2_ (Equations (2) and (3)): Fe^2+^ and Cu^+^ can induce the generation of hydroxyl radicals (·OH) and superoxide radicals (·O_2_^−^) through electron transfer between CuFe_2_O_4_@GAC and H_2_O_2_ (Equations (2) and (4)). H_2_O_2_ forms ·OH either under the activation of the CuFe_2_O_4_@GAC catalyst or by acquiring electrons; at the same time, Fe^2+^ acts with H_2_O_2_ to generate Fe^3+^ and ·OH (Equation (4)), and then Fe^3+^ further acquires electrons to convert to Fe^2+^. This process continuously provides reaction conditions for the degradation of Bisphenol A. The cyclic conversion of Fe^3+^/Fe^2+^ and Cu^+^/Cu^2+^ enables CuFe_2_O_4_@GAC to continuously activate H_2_O_2_ (Equations (4) and (5)), leading to the sustained generation of ·OH and ^1^O_2_ in the channel structure of CuFe_2_O_4_@GAC. Moreover, the cyclic regeneration of Fe^2+^/Fe^3+^ and Cu^+^/Cu^2+^ redox pairs is achieved through the reduction of Fe^3+^ and Cu^2+^. Benefiting from the efficient free radical generation capacity and favorable electron transfer characteristics of CuFe_2_O_4_@GAC, BPA is oxidized and mineralized more efficiently. Overall, the active free radicals of ·OH react with BPA molecules, ultimately mineralizing them into carbon dioxide (CO_2_) and water (H_2_O) (Equation (10)); the rapid cycling of Fe^3+^/Fe^2+^ and Cu^+^/Cu^2+^ not only improves the utilization efficiency of H_2_O_2_ but also inhibits carrier recombination. Combined with the results of X-ray photoelectron spectroscopy (XPS) analysis, it was seen that the ratio of Cu^+^/Cu^2+^ changed from 21.2% to 18.09%, and the ratio of Fe^3+^/Fe^2+^ decreased from 58.67% to 11.32%, indicating the conversion of Cu^+^/Cu^2+^ and Fe^3+^/Fe^2+^. This change directly confirms the cyclic conversion relationship between Fe^3+^/Fe^2+^ and Cu^+^/Cu^2+^ during the reaction, which is consistent with the next mechanism deduction.


(2) $$O_{2}+e^{-}\to O_{2}^{-}$$

(3) $$O_{2}+2H^{+}+2e^{-}\to H_{2}O_{2}$$

(4) $$Fe^{2+}+H_{2}O_{2}\to Fe^{3+}+\cdot \mathrm{OH}+OH^{-}$$

(5) $$Cu^{+}+H_{2}O_{2}\to Cu^{2+}+\cdot \mathrm{OH}+OH^{-}$$

(6) $$Cu^{2+}+H_{2}O_{2}\to Cu^{+}+\cdot OOH+H^{+}$$

(7) $$Cu^{2+}+e^{-}\to Cu^{+}$$

(8) $$Fe^{3+}+e^{-}\to Fe^{2+}$$

(9) $$Cu^{+}+Fe^{3+}\to Cu^{2+}+Fe^{2+}$$

(10) $$\cdot \mathrm{OH}+BPA\to {CO}_{2}+H_{2}O$$

The electrochemical performance of various modified glassy carbon electrodes (drop-casting method) was characterized using an electrochemical workstation via cyclic voltammetry (CV) and electrochemical impedance spectroscopy (EIS). The measurements were conducted in a mixed solution of 0.1 mol/L KCl and 5 mmol/L potassium ferricyanide/potassium ferrocyanide [Fe(CN)_6_]^3−^/^4−^.

**Figure 6:**
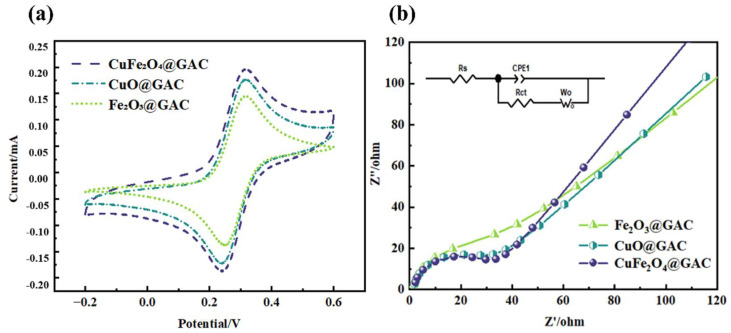
(**a**) CV curves of different catalysts in a 5 mmol L^−1^ K_3_[Fe(CN)_6_] (0.1 mol L^−1^ KCl) solution at a scan rate of 100 mV s^−1^. (**b**) EIS plots of different catalysts.

As shown in Figure 6, all catalytically modified electrodes clearly exhibited characteristic redox peaks of [Fe(CN)_6_]^3−^/^4−^ in this test system, indicating that effective charge transfer reactions could occur on the electrode surfaces: the oxidation peak currents of CuO@GAC, Fe_2_O_3_@GAC, and CuFe_2_O_4_@GAC (three metal oxide-loaded particle electrodes) were 1.76 × 10^−4^ A, 1.44 × 10^−4^ A, and 1.96 × 10^−4^ A, respectively. From the perspective of intrinsic electrochemical properties of materials, EIS test results (Figure 6b) showed that the diameter of the Nyquist arc corresponding to the CuFe_2_O_4_@GAC electrode was significantly smaller than that of the pure GAC electrode and the other two metal oxide@GAC composite electrodes. CuFe_2_O_4_@ GAC shows a lower charge transfer resistance (Table S5). This indicates enhanced charge transfer capability and lower electron transfer resistance at the electrode surface, and the electrodes have lower electron transfer resistance, making the charge transfer process at the electrode/electrolyte interface easier [33]. These results indicate that anchoring CuFe_2_O_4_ on GAC significantly reduces the overall electrode resistance. This change confirms that there is an obvious electronic interaction effect between the metal active component (CuFe_2_O_4_) and the carrier (GAC), which effectively optimizes the charge transfer efficiency of the electrode.

**Figure 7:**
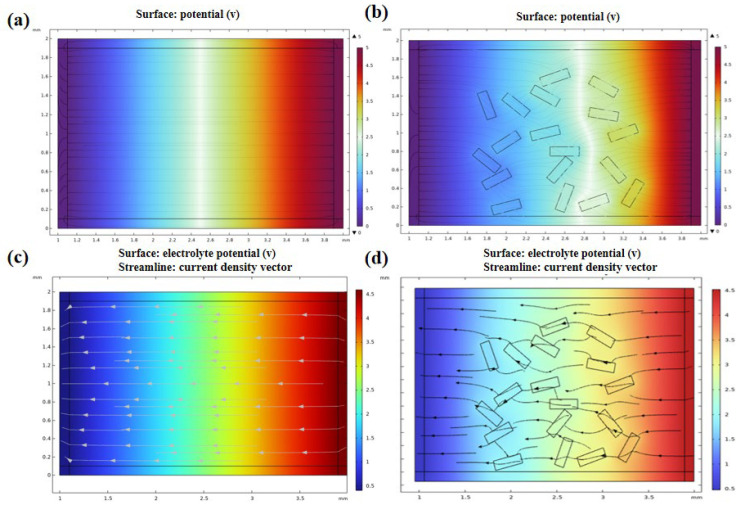
(**a**,**b**) Potential profile. (**c**,**d**) Electrolyte potential diagram and current density vector diagram.

The potential and current density distributions in the three-dimensional electro-Fenton system after adding the CuFe_2_O_4_@GAC particle electrodes were simulated via the COMSOL Multiphysics 6.4 software (Figure 7). As indicated by the simulation results, the introduction of CuFe_2_O_4_@GAC particle electrodes significantly altered the potential field distribution. The diversified potential gradient serves as the core factor for the formation of microelectrodes under the electric field effect [34,35,36]. A cross-particle potential gradient exists on the microelectrodes themselves, which directly regulates the electrochemical reactions occurring on their surface. A larger potential difference leads to more pronounced charge separation at the two ends of the particles, resulting in stronger anodic and cathodic polarity of the microelectrodes and greater reaction driving force for the formed “micro-electrolytic cells”.

### 3.4. Catalyst Stability and Application in Real Wastewater

Given that catalyst stability is crucial for electrode material performance, this study evaluated it through ion leaching, cyclic stability, and ion interference experiments. After the first reaction, the leaching concentrations of Fe and Cu were 4.756 mg/L and 1.630 mg/L, respectively. With the increase in the number of cycles, the leaching amount of both of them decreased significantly, to 0.769 mg/L and 0.216 mg/L, respectively, in the fifth cycle. The final stable concentrations of leached Cu (0.216 mg/L) and Fe (0.716 mg/L) ions are far below the surface water discharge standard of 2.0 mg/L stipulated by China and EU standards, demonstrating that the prepared catalyst possesses low metal leaching risk and favorable environmental safety [37]. The rapid decrease of leaching concentration is mainly due to the rapid dissolution of unstable metal components loaded on the catalyst surface in the initial reaction. In the subsequent cycle, the combination of active site and carrier is more stable, resulting in stable metal dissolution. The SEM characterization of the catalyst after five cycles showed that the loading of CuFe_2_O_4_ on the surface of activated carbon (GAC) decreased slightly, but most of the active components were still stably attached to the surface of the carrier. This also explains the main reason for catalytic deactivation: a small amount of copper and iron ions dissolved in the long-term reaction process, and the number of active sites decreased accordingly.

**Figure 8:**
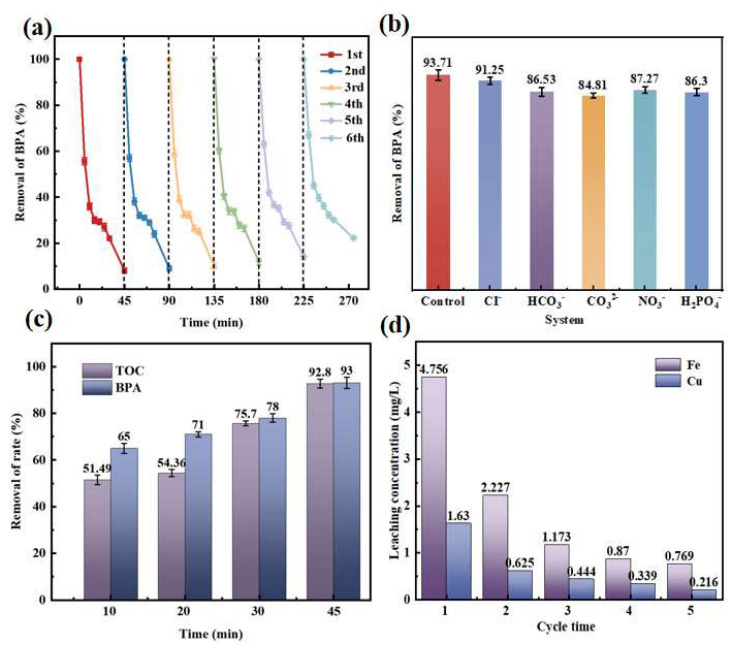
(**a**) Removing BPA after six recycling cycles. (**b**) Effects of inorganic anion. (**c**) TOC removal. (**d**) Metal leaching during successive reuse cycles.

Furthermore, the catalyst exhibits excellent cycling stability, maintaining a degradation rate of 86.2% even after five cycles. To investigate the interference effects of ubiquitous coexisting ions in actual water matrices (Figure 8b), typical anions including Cl^−^, HCO_3_^−^, CO_3_^2−^, NO_3_^−^ and H_2_PO_4_^−^ were selected for interference experiments, with the ultrapure water blank system (93.71% BPA removal efficiency) set as the reference group. The quantitative results showed that the five anions consumed the active free radicals of the system to varying degrees, thereby inhibiting BPA degradation. The quenching effect of CO_3_^2−^ was the strongest, and the corresponding degradation inhibition rate was 9.50%. The inhibition rates of H_2_PO_4_^−^ and HCO_3_^−^ were 7.91% and 7.66%, respectively. NO^3−^ brought 6.87% degradation loss. The Cl^−^ quenching effect was the weakest, only causing a decrease of 2.63% removal rate. HCO_3_^−^ and CO_3_^2−^ are the main ·OH radical quenching agents. Although Cl^−^ can also quench ·OH, the converted Cl· can still degrade BPA, so the inhibition effect is not obvious.


(11) $$\mathrm{MCE}(\%)=\frac{({\mathrm{TOC}}_{0}-{\mathrm{TOC}}_{t})\;V\;n\;F}{12000\;I\;t}\times 100\%$$

(12) $$\mathrm{EE}/O=\frac{P\cdot t}{V\cdot \log_{10}(C_{0}/C_{t})}$$

To investigate the mineralization ability of the system, the change trend of the BPA degradation rate and the TOC removal rate at different reaction times is shown in Figure 8c. The rapid degradation stage of TOC was 10 min before the reaction, and the TOC concentration decreased from the initial 17.46 mg/L to 8.47 mg/L, and the reaction rate was the fastest at this stage. With the extension of reaction time to 45 min, the TOC concentration further decreased to 1.62 mg/L, and the mineralization degree of organic matter increased continuously. The mineralization current efficiency and electrochemical energy consumption at different times were analyzed (Table S6). The mineralization current efficiency (MCE) showed a continuous downward trend with the increase in reaction time: the MCE reached 36.14% after 10 min of reaction, which was the highest value in the whole period, then at 20 min and 30 min, it decreased to 19.08% and 17.72%, respectively. At 45 min, the date of MCE was only 14.15%. This phenomenon is mainly due to the sufficient substrate concentration in the early stage of the reaction, the active free radicals acting on organic pollutants efficiently, and the conversion efficiency of electric energy to mineralization reaction being high. In the later stage, the properties of residual organic matter and intermediate products are more stable and the current utilization efficiency gradually decreases.

The energy consumption (EC) increases with the reaction time: the energy consumption is only 0.093 kWh/g at 10 min, and the energy consumption level is the best, while for the progress of the reaction, the energy consumption gradually increased to 0.175 kWh/g (20 min) and 0.189 kWh/g (30 min), and reached 0.236 kWh/g in 45 min. This shows that it is more difficult to degrade in the later stage of the reaction, and it takes more electricity to remove organic carbon per unit mass. Under the experimental conditions, the EE/O value is between 2.6 and 5.5 kWh/m^3^/order, and the total EE/O is 3.63 kWh/m^3^/order, indicating that the electrochemical oxidation process has good energy efficiency.

To evaluate practical application potential, four types of water matrices were selected as reaction media, including pure water, tap water, filtered lake surface water, and diluted BPA-containing dye wastewater. Pure water is taken from the laboratory water purifier, and only the target pollutant BPA is added. Tap water is taken from municipal domestic water in Hefei, and contains conventional trace inorganic ions. Lake water is taken from a Hubingtang water sample from Hefei University of Technology, including natural humus and other organic substances, as well as a variety of inorganic ions. The actual wastewater comes from the production wastewater of dye chemical plant, which contains Bisphenol A and various organic pollutants.

As displayed in Figure S5, the removal rate of BPA in pure water can reach more than 93%. When applied to the tap water system, the BPA removal rate remained above 92.9%, and the overall removal performance did not show significant attenuation. The degradation rate declines to 90.6% in lake water, while it reaches 89.2% in industrial wastewater. These results confirm the possibility of the system for actual wastewater remediation. The discrepancy originates from complex constituents in natural water matrices. Coexisting inorganic ions and natural organic matter can compete with BPA for reactive oxygen species and participate in side redox reactions, which suppress the oxidation process and ultimately reduce BPA degradation efficiency [38,39].

### 3.5. Degradation Pathway of BPA

**Figure 9:**
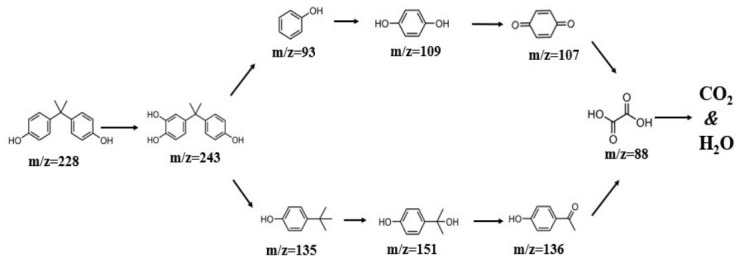
Degradation routes of BPA molecule in 3DEF-CuFe_2_O_4_@GAC.

Water samples with reaction times of 30 min and 45 min were used for ultra-performance liquid chromatography–mass spectrometry (UPLC-MS) detection. The degradation intermediates of BPA were identified. The structural formula, molecular formula, and mass-to-charge ratio (*m*/*z*) are shown in the attached table and Figure 9. According to the determined products, a reasonable degradation pathway of Bisphenol A can be proposed.

In the BPA molecule, the carbon atom adjacent to the hydroxyl group is prone to hydroxylation, addition, and oxidation reactions, leading to the introduction of a hydroxyl group onto the benzene ring and the formation of hydroxylated Bisphenol A. Subsequently, the bond is cleaved by an ·OH radical to form phenol (*m*/*z* = 93), which undergoes further hydroxylation and ring-opening to yield hydroquinone (*m*/*z* = 109) and p-benzoquinone (*m*/*z* = 107), and finally undergoes ring-opening to form oxalic acid (*m*/*z* = 88). Another pathway involves BPA breaking a bond to form tert-butylphenol (*m*/*z* = 135), which is further oxidized to tert-butyl alcohol (*m*/*z* = 151) and p-hydroxyacetophenone (*m*/*z* = 136) and finally undergoes ring opening to form oxalic acid, which is ultimately mineralized into CO_2_ and H_2_O [40,41].

### 3.6. Toxicity Assessment

**Figure 10:**
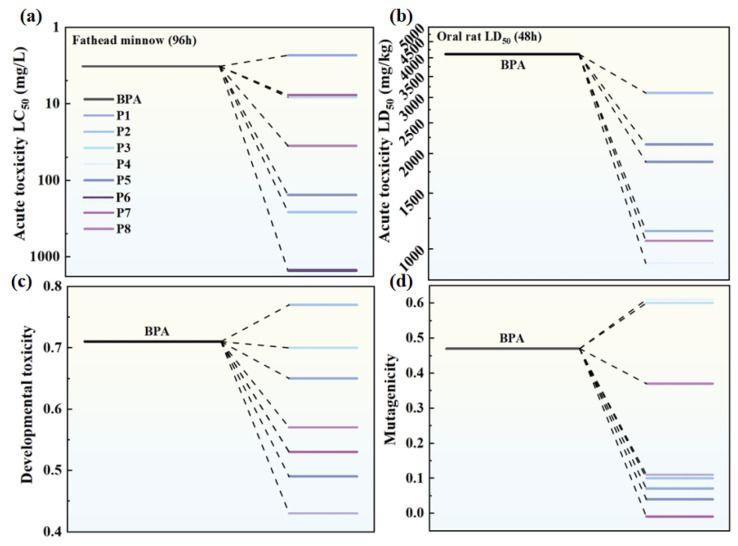
(**a**) Fathead minnow LC50 (96 h) of BPA and its degradation intermediates. (**b**) Oral LD50 of rats. (**c**) Developmental toxicity. (**d**) Mutagenicity.

The Toxicity Estimation Software Tool (TEST v5.1) enables the toxicity assessment of diverse molecular structures. Figure 10 presents four toxicological endpoints of BPA and its degradation intermediates, all derived via in silico simulation with the software. Following a 45 min reaction, the generated intermediates were subjected to TEST analysis to quantify four key endpoints, namely the rat oral median lethal dose (LD_50_), median lethal concentration (LC_50_), developmental toxicity potential, and mutagenicity of BPA and its derivatives.

All detected intermediates exhibited attenuated developmental toxicity relative to the parent compound (Figure 10c). Moreover, the final degradation products and the majority of intermediates displayed diminished mutagenicity compared with pristine BPA (Figure 10d). Collectively, these findings indicate that the ultimate products of BPA degradation pose a low environmental risk.

## 4. Conclusions

This study successfully prepared a novel bimetallic oxide-loaded GAC particle electrode (CuFe_2_O_4_@GAC), using granular activated carbon (GAC) as the support. The catalytic degradation performance of this catalyst for BPA was systematically investigated under optimal experimental parameters. The results showed that CuFe_2_O_4_@GAC had better H_2_O_2_ activation ability and could effectively degrade BPA. Combined with BET, XPS, and electrochemical characterization, CuFe_2_O_4_@GAC has a specific surface area and a more microporous structure, providing more active centers. It also has the lowest electron transfer resistivity and reduction potential and is more inclined to redox ability. Moreover, the highly efficient reversible cycles of Cu^2+^/Cu^+^ and Fe^2+^/Fe^3+^ significantly improved the stability and reusability of the catalytic system. Cycle performance tests demonstrated that the CuFe_2_O_4_@GAC particle electrode maintained stable degradation efficiency after five consecutive catalytic cycles, showing good potential for practical application. This study provides an important theoretical basis and technical reference for the design and development of high-efficiency electro-Fenton catalysts and the expansion of their applications in the field of organic pollutant degradation.

## Figures and Tables

**Table 1:** Performance comparison of three-dimensional particle electrodes.

Catalyst	Pollutant	Removal Efficiency (%)	Time (min)	Initial Concentration (mg/L)	pH	TOC/COD	Energy Consumption	Stability (Cycles)	References
CuFe_2_O_4_/Al_2_O_3_	p-nitrophenol (PNP)	90.69%	30	150	10	—	300 kWh/kg	—	[14]
GAC and PCP	Heavy oil refinery wastewater	30.8%	55	2973	7	43.3%	53.65 kWh/kg	10	[15]
TMP	BPA	>98%	55	10	9	—	—	4 (>90%)	[16]
GAC	Paper mill wastewater	45%	60	1357	11	—	910 kWh/kg	—	[17]
Steel Slag	Rhodamine B	100%	60	5	6	—	0.15 kWh/L	—	[18]
γ-Al_2_O_3_	Tetracycline	86%	180	100	5.9	—	—	10	[19]
Ceramic particle	Rhodamine B	83.45%	150	750	3	76.9%	—	—	[20]
CuFe_2_O_4_@GAC	BPA	93%	45	20	3	92.8%	23.67 kWh/kg	6 (>77%)	This work

## Data Availability

The original contributions presented in this study are included in the article/Supplementary Material. Further inquiries can be directed to the corresponding authors.

## Supplementary Materials

The following supporting information can be downloaded at https://www.mdpi.com/article/10.3390/nano16120722/s1, Figure S1: (a) SEM image of GAC, (b) SEM image of CuO @GAC, (c) SEM image of Fe2O3@GAC, (d–f) SEM morphologies of CuFe_2_O_4_@GAC samples with varying Cu/Fe molar ratios, (g–i) HRTEM image and element mapping image of CuFe_2_O_4_@GAC after the cyclic experiment; Figure S2: EDS spectrum of the as-synthesized CuFe_2_O_4_ sample; Figure S3: XRD patterns of different molar ratios of Cu/Fe; Figure S4: (a,b) XPS spectra of O 1s before and after reaction, (c,d) XPS spectra of Survey before and after reaction; Table S1: Conditions for liquid phase detection of BPA; Table S2: Specific surface area of CuO@GAC, Fe_2_O_3_@GAC, CuFe_2_O_4_@GAC and GAC; Figure S5: Analysis of real water samples; Table S3: The information on possible intermediates during the degradation of BPA; Figure S6: Liquid-mass diagram of BPA and its intermediates in 3D-EF system. Table S4: Comsol simulation parameters; Figure S7: Schematic diagram of degradation mechanism; Figure S8: SEM image of particle electrode after 5 cycles; Table S5: Simulated parameters of the elements in equivalent circuits of Particle electrode; Table S6: MCE, energy consumption and TOC at different time; Table S7: Electrical Energy per Order; Table S8: Water quality parameters of actual water samples.
