# Supplementary material for: Three-Dimensional Electro-Fenton System with CuFe2O4-Loaded Granular Activated Carbon as the Catalytic Particle Electrode for Removal of Bisphenol A
Source: Nanomaterials (Basel). 2026 Jun 11;16(12):722. doi: 10.3390/nano16120722 (PMC13304731; doi:10.3390/nano16120722)
Supplement: Supplementary file 1 [file nanomaterials-16-00722-s001.zip › nanomaterials-4342945-supplementary.pdf]

Supporting Information

Three-Dimensional Electro-Fenton System with $CuFe_2O_4$-Loaded Granular Activated Carbon as the Catalytic Particle Electrode for Bisphenol A Removal

Sheng Tao [1,2,†], Zhang Luo [3,†], Defeng Kong [4], Yifan Chai [1,2], Shenglong Kuai [5], Huilai Liu [5], Cheng Yin [5,*] and Xing Chen [1,2,*]

1 School of Resources and Environmental Engineering, Hefei University of Technology, Hefei 230009, China;

2 Key Lab of Aerospace Structural Parts Forming Technology and Equipment of Anhui Province, Institute of Industry and Equipment Technology, Hefei University of Technology, Hefei 230009, China

3 CCCC Yangtze River Construction Development Group Co., Ltd., Chongqing 400700, China;

4 Anhui Haoyue Ecological Technology Co., Ltd., Hefei 230071, China;

5 School of Resource and Environmental Engineering, Anhui Water Conservancy Technical College, Hefei 231603, China;

* Correspondence: yc@ahsdxy.edu.cn (C.Y.); xingchen@hfut.edu.cn (X.C.)

† These authors contributed equally to this work.

Outlines

Figure S1. (a) SEM image of GAC, (b) SEM image of CuO @GAC, (c) SEM image of Fe_2O_3@GAC, (d-e) SEM morphologies of $CuFe_2O_4$@GAC samples with varying Cu/Fe molar ratios, (g-i) HRTEM image and element mapping image of $CuFe_2O_4$@GAC after the cyclic experiment.

Figure S2. EDS spectrum of the as-synthesized $CuFe_2O_4$ sample.

Figure S3. XRD patterns of different molar ratios of Cu/Fe.

Figure S4. (a-b) XPS spectra of O 1s before and after reaction, (c-d) XPS spectra of Survey before and after reaction.

Table S1. Conditions for liquid phase detection of BPA.

Table S2. Specific surface area of CuO@GAC, Fe_2O_3@GAC, $CuFe_2O_4$@GAC and GAC.

Figure S5. Analysis of real water samples.

Table S3. The information on possible intermediates during the degradation of BPA.

Figure S6. Liquid-mass diagram of BPA and its intermediates in 3D-EF system.

Table S4. Comsol simulation parameters.

Figure S7. Schematic diagram of degradation mechanism.

Figure S8. SEM image of particle electrode after 5 cycles.

Table S5. Simulated parameters of the elements in equivalent circuits of Particle electrode.

Table S6. MCE, energy consumption and TOC at different time.

Table S7. Electrical Energy per Order.

Table S8. Water quality parameters of actual water samples.

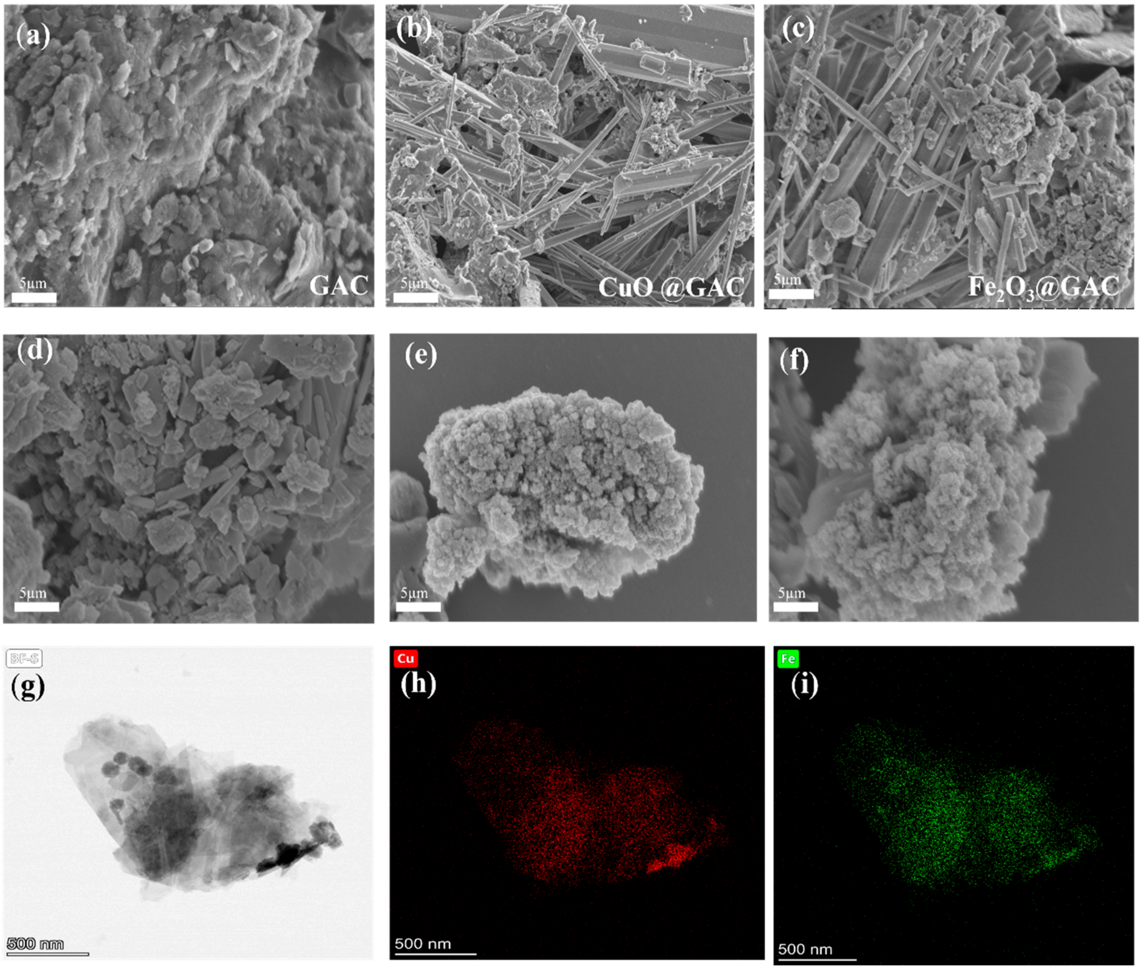

Figure S1. (a) SEM image of GAC, (b) SEM image of CuO@GAC, (c) SEM image of Fe_2O_3@GAC, (d-e) SEM morphologies of $CuFe_2O_4$@GAC samples with varying Cu/Fe molar ratios(n(Cu):n(Fe)=1:1;1:2;1:3), (g-i) HRTEM image and element mapping image of $CuFe_2O_4$@GAC after the cyclic experiment.

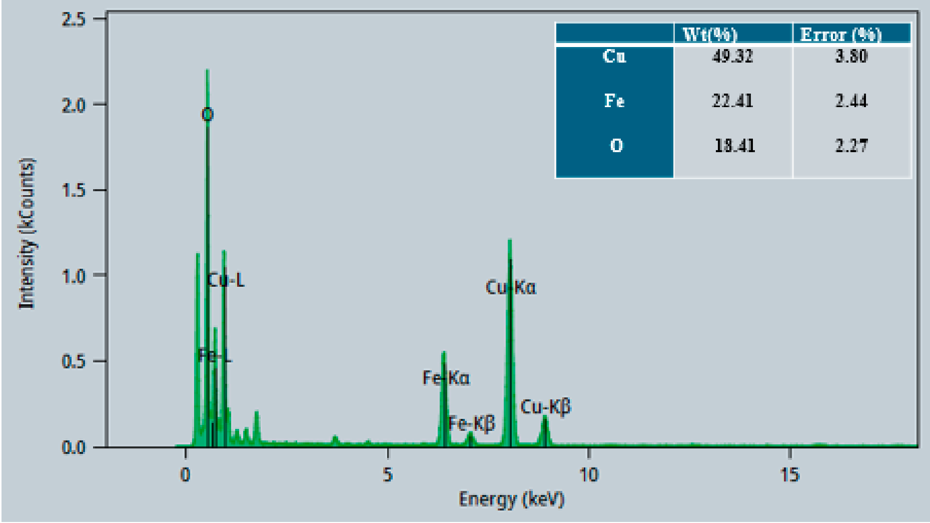

Figure S2. EDS spectrum of the as-synthesized $CuFe_2O_4$ sample.

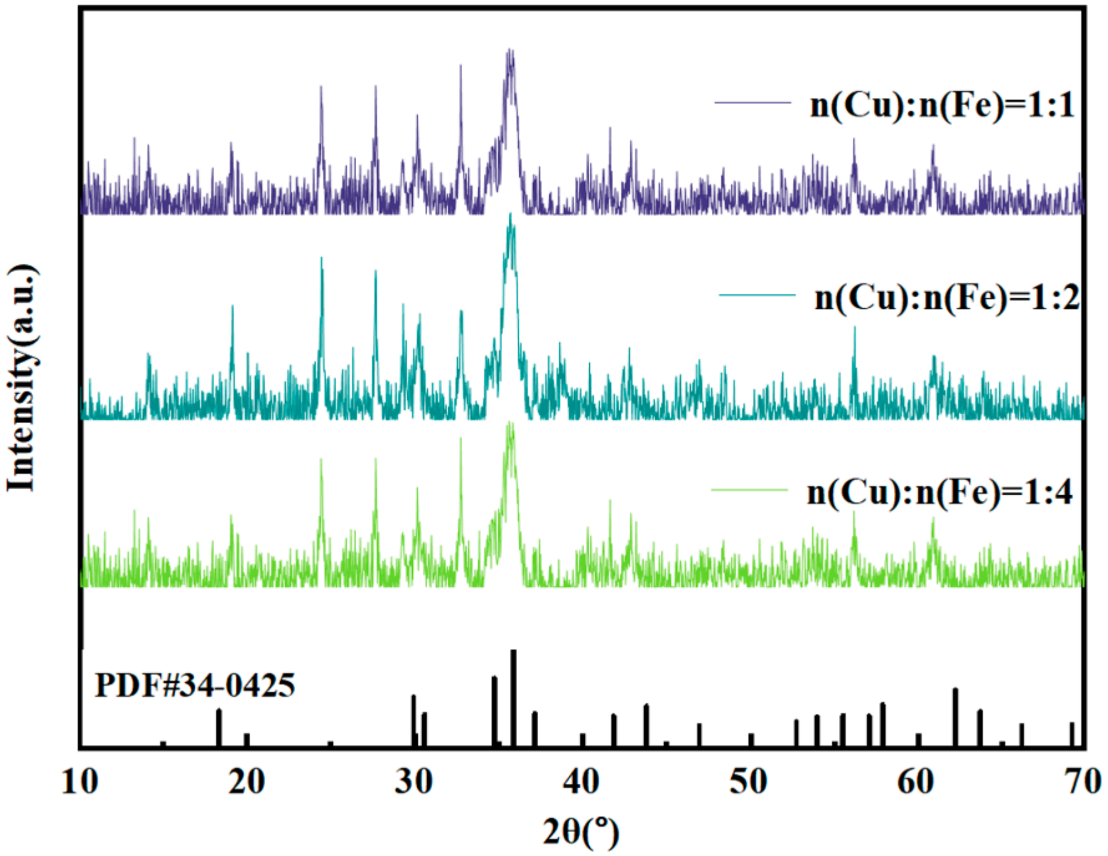

Figure S3. XRD patterns of $CuFe_2O_4$ samples prepared with different n(Cu):n(Fe) molar ratios.

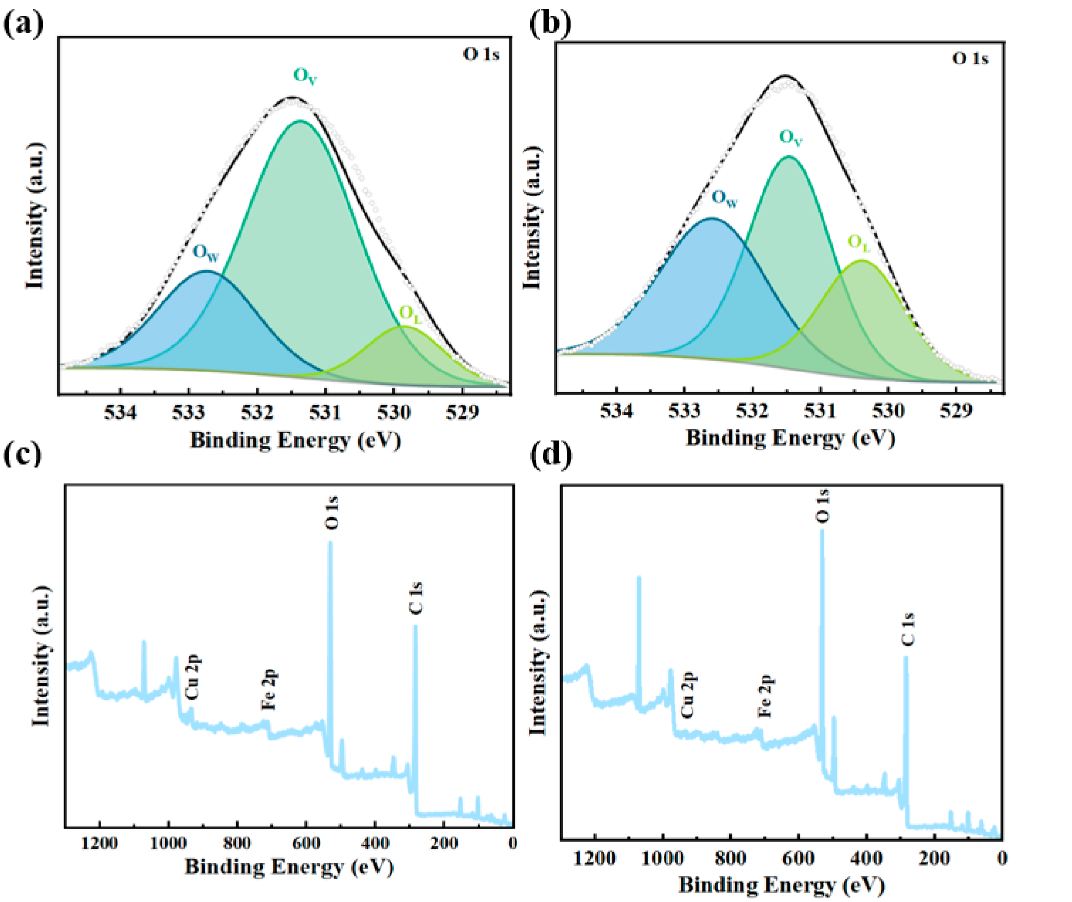

Figure S4. (a-b)XPS spectra of O 1s before and after reaction, (c-d)XPS spectra of Survey before and after reaction.

Table S1. Conditions for liquid phase detection of BPA.

Compounds	Mobile phase	Detection wavelength (nm)	Retention time (min)
BPA	30% water + 70% methanol	230	4.5 ± 0.1

Table S2. Specific surface area of CuO@GAC, Fe_2O_3@GAC, $CuFe_2O_4$@GAC and GAC.

Samples	S_{BET} (m^2g^{-1})	Pore volume(cm^3g^{-1})	Pore diameter (nm)
CuO@GAC	31.646	0.085	3.060
Fe_2O_3@GAC	30.218	0.076	3.810
$CuFe_2O_4$@GAC	39.995	0.020	3.818
GAC	31.946	0.012	3.828

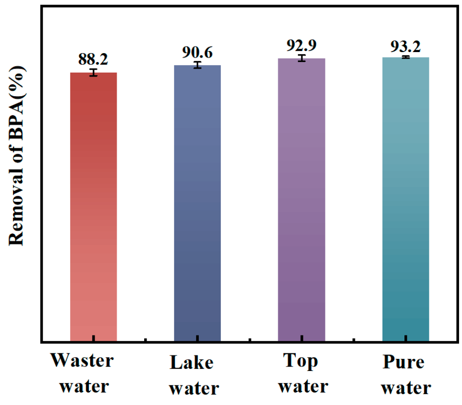

Figure S5. Analysis of real water samples.

Table S3. The information on possible intermediates during the degradation of BPA.

	Formula	Extracted m/z	Chemical structure
BPA	$C_{15}H_{16}O_2$	227	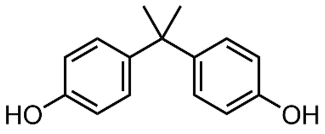
P1	$C_{15}H_{16}O_3$	243	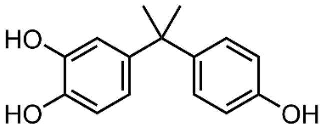
P3	C_6H_6O	93	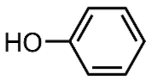
P4	$C_{10}H_{14}O$	135	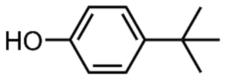
P5	$C_3H_6O_3$	88	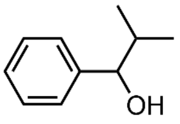
P6	$C_6H_4O_2$	107	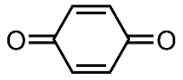
P7	$C_6H_6O_2$	109	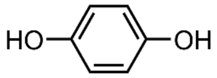
P8	$C_9H_{12}O_2$	151	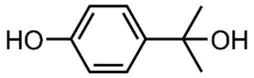
P9	$C_8H_8O_2$	136	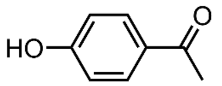

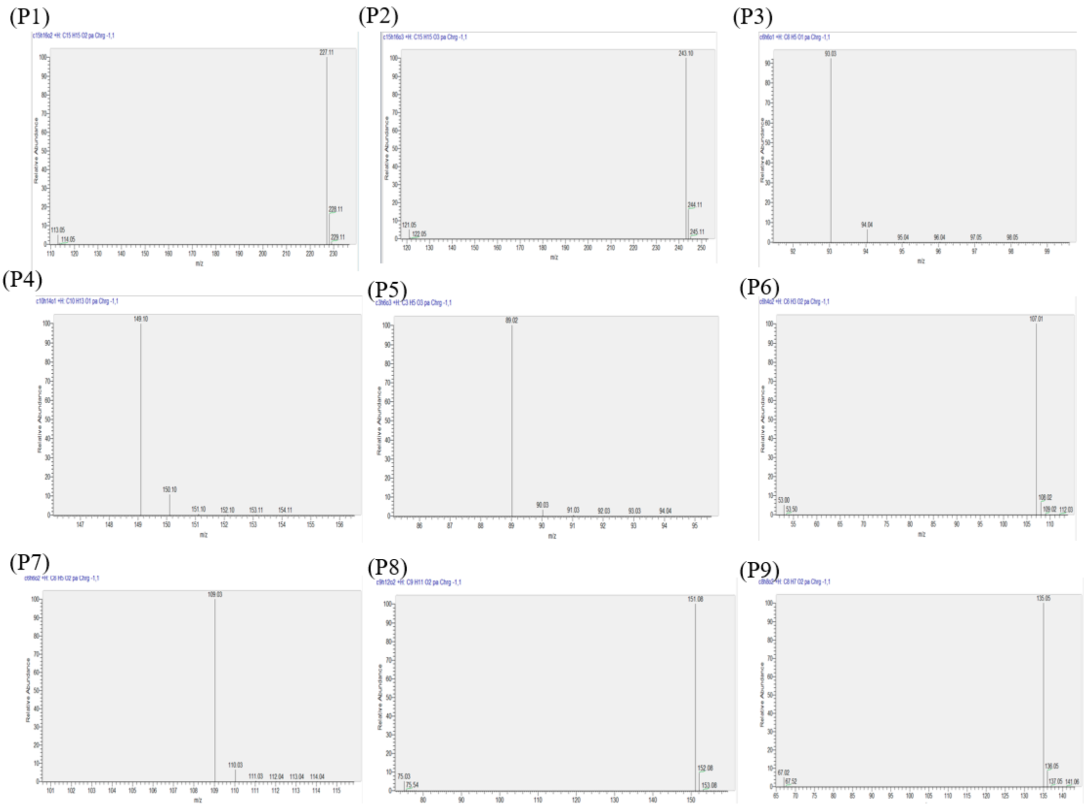

Figure S6. Liquid-mass diagram of BPA and its intermediates in 3D-EF system.

Table S4. Comsol simulation parameters.

	Geometric parameter(mm)	Initial voltage(v)	Electrical conductivity(σ)	Relative dielectric Constant(ε_r)
Particle electrode	1×4×1mm	—	15 S/m	3
Graphite sheet electrode	4×18×1mm	0/5V	120 S/m	5
electrolyte solution	—	—	1.1 S/m	80
electrolytic bath	20×30×20mm	—	—	—
Physical field coupling	Secondary current distribution+current module			

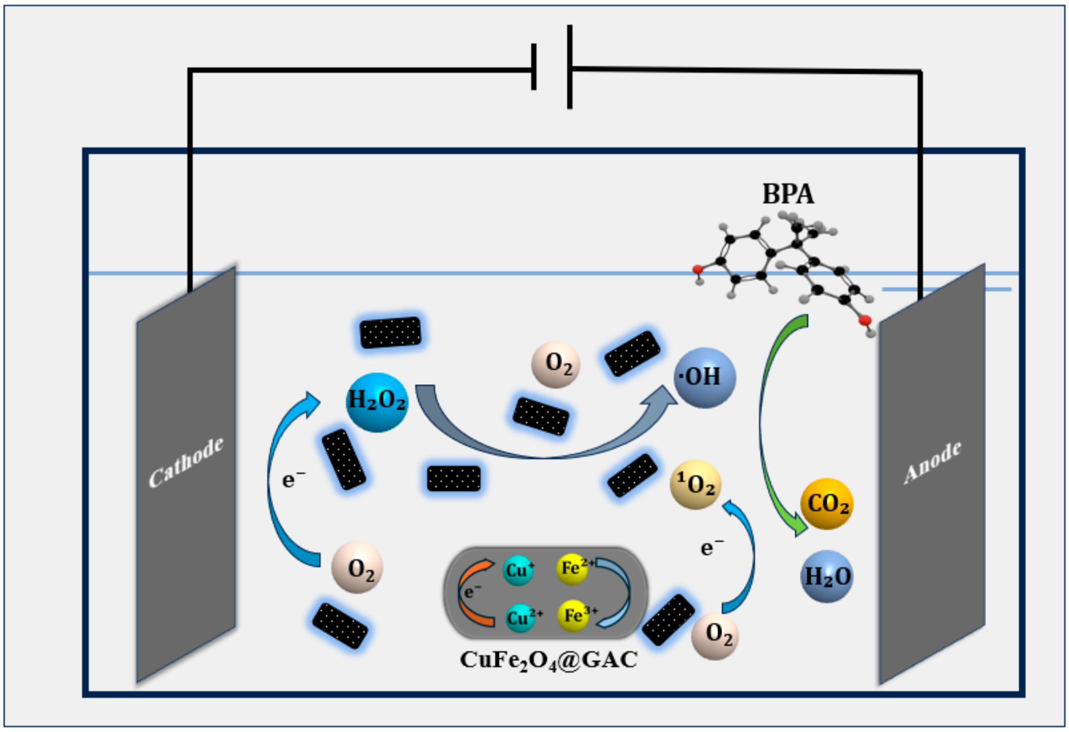

Figure S7. Schematic diagram of degradation mechanism.

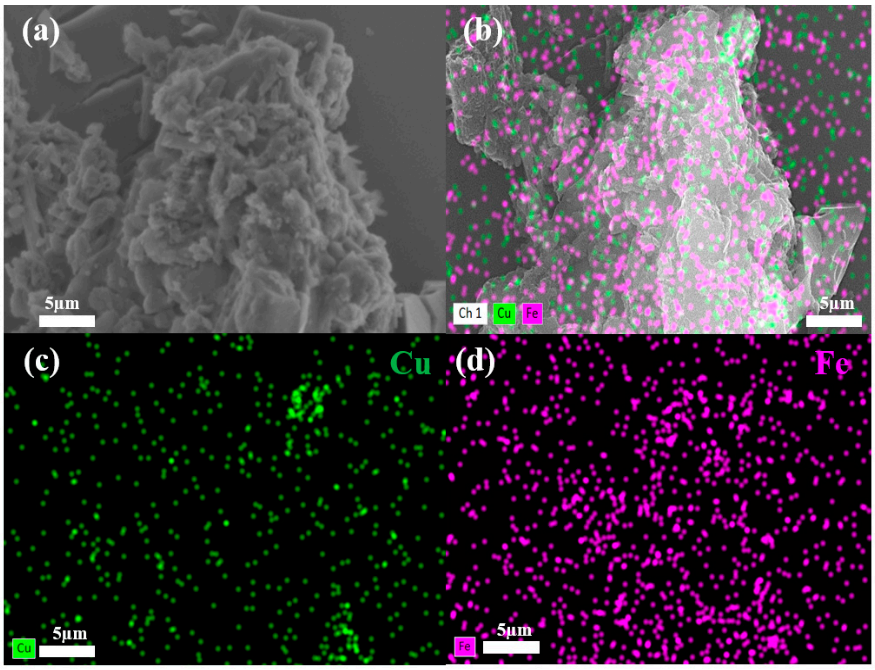

Figure S8. SEM image of particle electrode after 5 cycles.

Table S5. Simulated parameters of the elements in equivalent circuits of Particle electrode.

Element	CuO@GAC		Fe_2O_3@GAC		$CuFe_2O_4$@GAC	
	Value	Error%	Value	Error%	Value	Error%
$R_s(\Omega)$	1.37	4.89	0.89	14.38	1.24	15.57
CPE-T(F)	1.98E-6	9.95	3.65E-6	19.02	2.43E-6	20.62
CPE-P(F)	0.93	0.94	0.89	1.89	0.91	2.05
$R_{ct}(\Omega)$	40.01	3.83	52.3	5.93	38.03	3.79

Table S6. MCE, energy consumption and TOC at different time.

**Time(min)**	**TOC(mg/L)**	**MCE(%)**	**EC(kWh/g $_{TOC}$)**

0	17.46	—	—
10	8.47	36.14	0.093
20	7.97	19.08	0.175
30	4.24	17.72	0.189
45	1.62	14.15	0.236

Table S7. Electrical Energy per Order.

**Reaction time (min)**	**TOC removal (%)**	**log (C_0/C)**	**EE/O (kWh/m^3/order)**
0-10	51.5	0.314	2.65
10-30	54.4	0.301	5.55
30-45	75.7	0.418	2.99
Overall	92.8	1.033	3.63

Table S8. Water quality parameters of actual water samples.

**Water body type**	**COD(mg/L)**	**pH**	**Conductivity (μS/cm)**	**Concentration (mg/L)**
**Lake water**	175	7.2	183	n.d.
**Waste water**	2023	9.5	6170	13.25
**Tap water**	4.53	7.1	191	n.d.
